# Experimental and Numerical Study of Concrete Fracture Behavior with Multiple Cracks Based on the Meso-Model

**DOI:** 10.3390/ma16186311

**Published:** 2023-09-20

**Authors:** Zhanliang Wang, Wei Zhang, Yiqun Huang

**Affiliations:** School of Civil Engineering, Fujian University of Technology, Fuzhou 350118, China; wangzl2012@fjut.edu.cn (Z.W.);

**Keywords:** concrete, double cracks, meso-model, cohesive element

## Abstract

In this paper, a series of experimental and numerical studies were carried out to investigate the effect of multiple cracks on concrete fracture behavior. Seven groups of double-crack concrete three-point bending (TPB) experiments with different crack lengths and different crack distances were carried out. The experimental results showed that the bearing capacity of double-crack specimens was slightly larger than the standard specimen with one central crack. Additionally, with an increase in the second crack length or with a crack distance reduction, the concrete’s bearing capacity increased correspondingly. Based on the experiments, a numerical meso-model was developed based on applying cohesive elements. The aggregate, mortar, interface transition zone (ITZ), and potential fracture surfaces were explicitly considered in the model. In particular, cohesive elements were used to characterize the mechanical behavior of the ITZ and potential fracture surfaces. A modified constitutive concrete model was developed by considering the potential fracture surfaces’ damage relation and friction effect. The accuracy of the developed meso-model was validated through a comparison between simulation and experiments. Based on meso-models, the influence of multiple cracks on the concrete bearing capacity was investigated by analyzing the energy evolution. The analysis results showed that the bearing capacity has a linear relation with the proportion of mode II energy consumption during the fracture process, which explains why specimens with multiple cracks have a slightly larger bearing capacity than the standard specimens. In summary, this study has found that in three-point bending fracture tests primarily characterized by mode I fractures, the presence of multiple cracks near the main crack slightly enhances the load-bearing capacity of the specimens. This is attributed to a slight increase in internal energy dissipation associated with the presence of these multiple cracks.

## 1. Introduction

Concrete’s fracture performance has a significant impact on the safety of bridges or other structures. When concrete cracks, it can affect the corrosion resistance of the bridge and its corresponding load-bearing capacity [1,2,3,4]. Therefore, it is essential to conduct detailed research on the fracture performance of concrete.

Since the theory of fracture mechanics was first used to analyze the failure behavior of concrete, a series of research works on concrete fracture mechanics have been carried out. Researchers studied the influence of the size effect [1,2,3,4,5,6,7], the loading rate [8,9], cyclic loading [10], and corrosion [11] (and so on) on concrete fracture behavior. The fracture mechanics approach can also be used to analyze complex and composite concrete structures, such as geopolymer concrete [12], reinforced concrete [13], fiber-reinforced concrete [14], and recycled concrete [15].

In practical engineering, concrete may have multiple cracks due to the construction technology and natural factors, and these cracks often affect each other. However, there are only a few papers about the fracture behavior of concrete with multiple cracks [16]. Evaluating or analyzing the bearing capacity of concrete structures with multiple cracks is still complex.

Due to the complexity of the multiple crack problem, it is hard to analyze the fracture behavior of concrete with multiple cracks using the mathematical analysis method. Thus, the mesoscopic numerical method is more suitable for studying this problem. Up to now, many mesoscopic methods have been proposed, such as the traditional finite element method [17,18,19], the lattice model [20,21,22], the extended finite element method [23,24,25], the particle flow method [26,27], the rigid body spring method [28], and the cohesive zone model (CZM) [29,30,31,32,33,34]. In particular, based on the application of cohesive elements, the CZM model is among the most advantageous numerical methods for characterizing interfacial mechanical behavior. For this reason, this kind of model can be used to simulate the fracture behavior of concrete fracture surfaces since it can accurately characterize cracks along non-prescribed trajectories.

This study conducted a series of TPB experiments to investigate the effect of multiple cracks on the mode I fracture behavior. Double-crack TPB beams with different second crack lengths and different crack distances were designed and tested. In addition, a meso-model was developed based on the CZM and the corresponding constitutive model. Finally, based on the experimental results and the developed meso-model, the fracture process and the inner energy consumption were analyzed.

## 2. Three-Point Bending (TPB) Experiments of a Double-Crack Concrete Beam

### 2.1. Geometry and Loading Scheme

**Figure 1:**
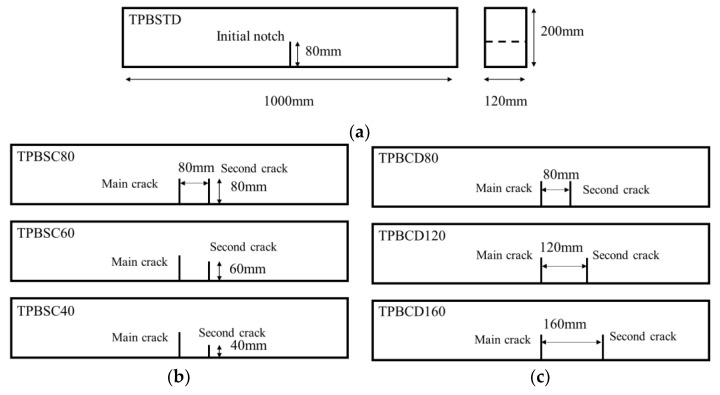
Design of concrete double-crack beams: (**a**) standard TPB beam; (**b**) beams with different second crack lengths; (**c**) beams with different crack distances.

To compare with the standard mode Ⅰ fracture behavior of concrete, double-crack TPB concrete beams were designed based on previous works [35]. The standard TPB beams with an initial crack length of 80 mm were chosen as the reference group, and the size of this kind of beam is length (l) × height (h) × thickness (t) = 1000 mm × 200 mm × 120 mm, as shown in Figure 1a. Based on the standard specimen, two series of experiments were designed to study the effect of second crack length (40 mm, 60 mm, 80 mm) and the crack distance (80 mm, 120 mm, 160 mm) on the fracture behavior of concrete beams; the corresponding sizes of the specimens are shown in Figure 1b,c. The design information of the experiments is listed in Table 1. In particular, TPBSTD represents the standard TPB beam, TPBSC represents the double-crack beams with different second crack lengths, and TPBCD represents the double-crack beams with different crack distances. 

In every experimental group, four specimens were cast to eliminate random errors, and all beams were cast at once. A steel plate with a thickness of 3 mm was used to make the initial cracks. The particle size of aggregates was distributed in the range of 5~20 mm continually. P.O 42.5 cement, sand, and water were chosen to cast the mortar matrix of the concrete. The mix proportion is cement:aggregate:sand:water = 1:1.225:2.458:0.44. Through the material property tests, the standard compressive strength, tensile strength, and elastic modulus were determined to be about 49 MPa, 3.5 MPa, and 45 GPa, respectively.

**Figure 2:**
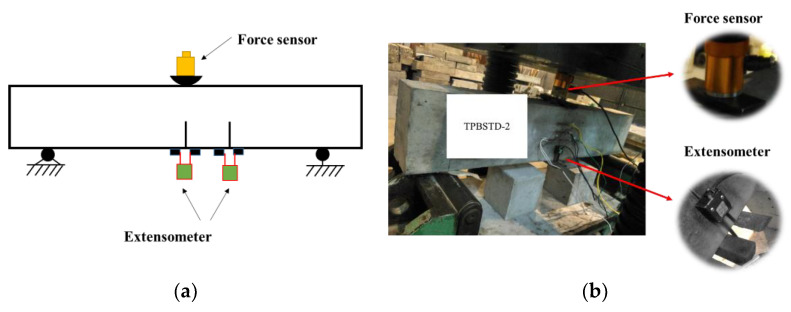
Loading scheme of the double-crack beams: (**a**) designed loading scheme; (**b**) experimental situation.

The loading scheme of the beams is shown in Figure 2. The distance between the two supports is 800 mm, and two clip-on extensometers with a 2 mm range were used to record the crack mouth opening displacement (CMOD) of the two cracks. Additionally, a force sensor with a 50 kN range was used to record the loading value. The experiments were carried out on a 500 t hydraulic testing machine through the displacement control method, and the loading rate was set to about 1.5 × 10^−3^ mm/s. All measured parameters were recorded through a DH-5902 testing system (sampling frequency: 10 to 100 k Hz).

### 2.2. Experimental Results

**Figure 3:**
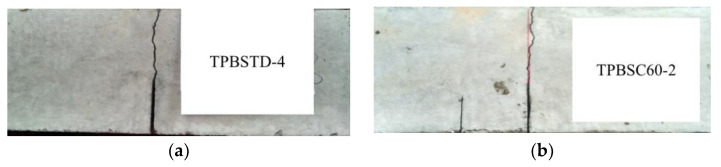
Typical fracture behavior of a concrete specimen: (**a**) the typical fracture behavior of a standard specimen; (**b**) the typical fracture behavior of a double-crack specimen.

The typical fracture pattern of the TPB beams is shown in Figure 3. All the crack propagation paths of the double-crack TPB beams were almost the same as the standard TPB beams, and the second crack was not observed to propagate at the macro-scale. This result indicated that in the failure process, which is dominated by mode I fracture, the second crack hardly affected the crack propagation path of the main crack (middle crack) when the length of the second crack was less than the main crack.

**Figure 4:**
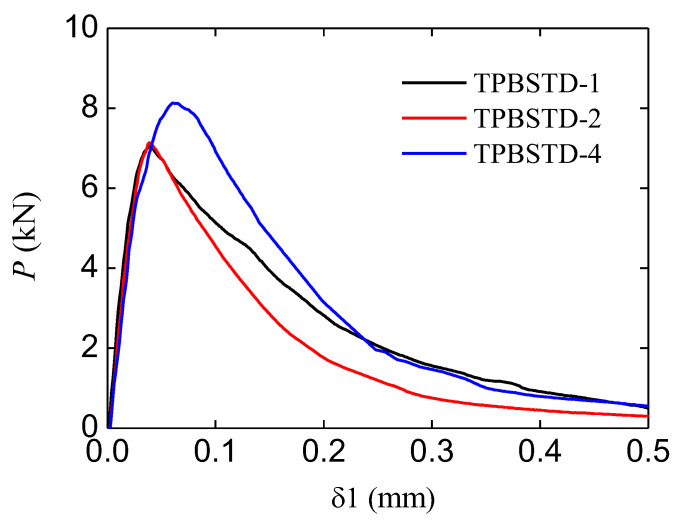
$P\sim \delta_{1}$ curve of standard specimens.

In this study, the CMOD of the main crack was called $\delta_{1}$, and that of the second crack was called $\delta_{2}$. In addition, the load value obtained from the force sensor was represented by *P*. The typical $P-\delta_{1}$ curves of standard TPB beams are shown in Figure 4. The curves both showed an ascending branch and a softening branch. The average peak force of these cures is 7.38 kN, and the corresponding CMOD is 0.0462 mm.

**Figure 5:**
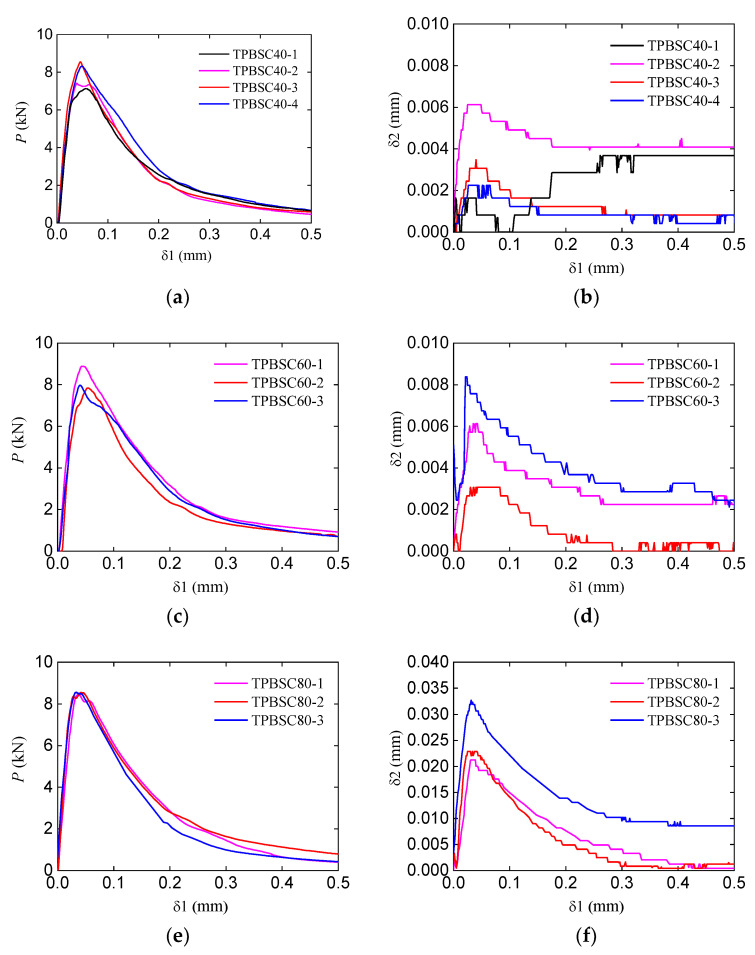
Experimental curves of double-crack beams with different second crack lengths: (**a**,**c**,**e**) $P-\delta_{1}$ curve with second crack lengths of 40 mm, 60 mm, and 80 mm; (**b**,**d**,**f**) $\delta_{2}\sim \delta_{1}$ curves with second crack lengths of 40 mm, 60 mm, and 80 mm.

Figure 5 shows the $P-\delta_{1}$ curves and $\delta_{2}-\delta_{1}$ curves of the corresponding double-crack TPB beams with different second crack lengths. Compared to standard beam results, the double-crack beam peak loads were slightly larger. In addition, with the increase in the second crack length, the bearing capacity of the beams increased somewhat. As for the CMOD of the second crack, $\delta_{2}$ increased with the increase in the loading force at first. After the loading force reached peak value, $\delta_{2}$ gradually decreased while the CMOD of the main crack $\delta_{1}$ continually increased. This result indicates that the second crack only propagated in the stable fracture stage (before the load reached peak value). Additionally, with the increase in second crack length, the value of the corresponding CMOD $\delta_{2}$ became larger. It should be noted that during the test, the increase in the CMOD $\delta_{2}$ may be smaller than the sensitivity of the extensometer; the $\delta_{2}-\delta_{1}$ curves may appear not as smooth as others, as shown in Figure 5b,d.

**Figure 6:**
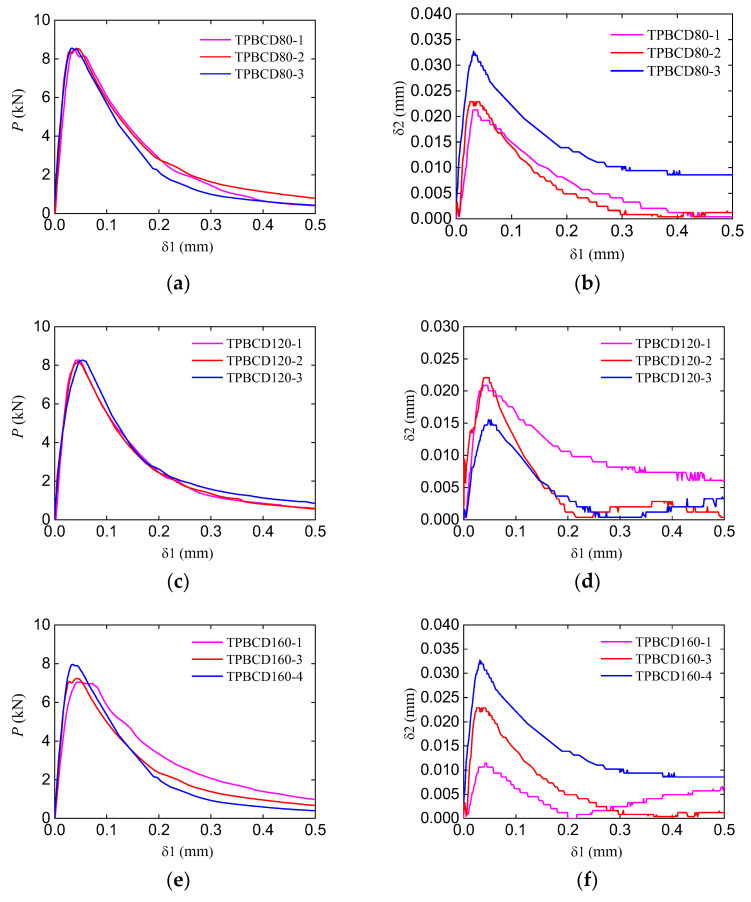
Experimental curves of the double-crack beams with different crack distances: (**a**,**c**,**e**) $P\sim \delta_{1}$ curves with crack distances of 80 mm, 120 mm, and 160 mm; (**b**,**d**,**f**) $\delta_{2}\sim \delta_{1}$ curves with crack distances of 80 mm, 120 mm, and 160 mm.

Figure 6 shows the $P-\delta_{1}$ curves and $\delta_{2}-\delta_{1}$ curves of the corresponding double-crack TPB beams with different crack distances. Similar to the beams with different second crack lengths, the bearing capacity of the beams with varying crack distances was slightly larger than the standard beams. In addition, with the decrease in the crack distance, the bearing capacity increased slightly, and the corresponding CMOD of the second crack $\delta_{2}$ increased. This result indicates that the influence of the second crack is inversely related to the crack distance between the two cracks.

To conveniently compare the influence of the second crack length and crack distance on the bearing capacity of the beams, the peak load and the corresponding CMODs were extracted and shown in Table 2. It should be noted that the group TPBSC80 is also called TPBCD80.

## 3. Establishment of a Numerical Model Based on the Cohesive Zone Model

### 3.1. Meso-Modeling Method

**Figure 7:**
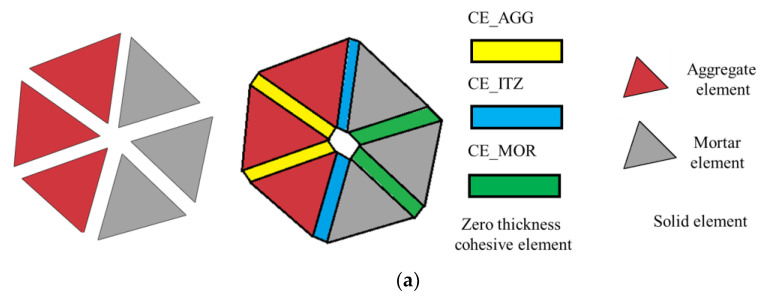

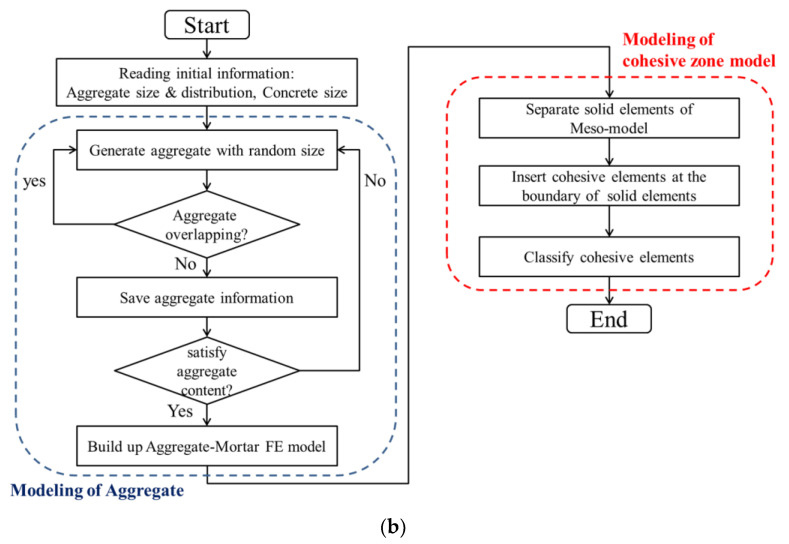
Meso-modeling of the concrete: (**a**) elements of the meso-model; (**b**) flowchart of modeling.

The meso-modeling method proposed earlier [32,33] was adopted to analyze the fracture behavior of concrete with double parallel cracks. This method characterized the potential fracture surfaces and ITZ via zero-thickness cohesive elements. These cohesive elements were inserted into all the interfaces of solid elements, as shown in Figure 7a. There are three kinds of cohesive elements in the numerical method: (1) CE_AGG for the potential fracture surfaces of aggregate; (2) CE_MOR for the potential fracture surfaces of mortar; and (3) CE_ITZ for the ITZ of concrete. The flowchart of meso-modeling [31,32] is shown in Figure 7b; the modeling method can be divided into two steps, which are the modeling of aggregates and modeling of the cohesive zone.

### 3.2. Constitutive Model of Concrete Potential Fracture Surfaces

(1) Single-mode damage relation

**Figure 8:**
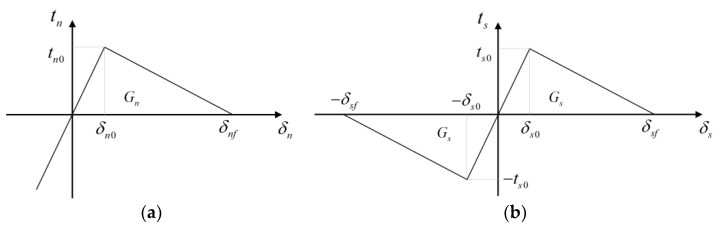
Bilinear relation of the concrete in pure normal or shear damage conditions: (**a**) in the normal direction; (**b**) in the tangential direction.

The modified constitutive model [32,33] based on the bilinear damage relation was adopted to characterize the mechanical behavior of the concrete’s potential fracture surfaces. The developed constitutive model especially considered the friction effect inside the crack. Figure 8 shows the damage relations of the concrete potential fracture surfaces in the normal and tangential directions. The expression of the stresses in the normal direction can be given as follows:

(1) $$t_{n}=\left\{\begin{array}{rr} k_{n}\delta_{n} & \delta_{n}\leq \delta_{n0}\\ (1-D)k_{n}\delta_{n} & \delta_{n0}<\delta_{n}<\delta_{nf}\\ 0 & \delta_{n}>\delta_{nf} \end{array}\right.$$

where $t_{n}$ is the normal stress; $k_{n}$ is the stiffness in the normal direction; $\delta_{n}$ is the normal displacement; $\delta_{n0}$ is the normal damage initiation displacement; $\delta_{nf}$ is the normal failure displacement; and D is the damage factor, which can be calculated through $D=\frac{(\delta_{n}-\delta_{n0})\delta_{nf}}{(\delta_{nf}-\delta_{n0})\delta_{n}}\;$.

The damage relation in the tangential direction can also be similarly given as follows:

(2) $$t_{s}=\left\{\begin{array}{rr} k_{s}\delta_{s} & \left|\delta_{s}\right|\leq \delta_{s0}\\ (1-D)k_{s}\delta_{s} & \delta_{s0}<\left|\delta_{s}\right|<\delta_{sf}\\ 0 & \left|\delta_{s}\right|>\delta_{sf} \end{array}\right.$$

where $t_{s}$ is the shear stress; $k_{s}$ is the stiffness in the tangential direction; $\delta_{s}$ is the tangential displacement; $\delta_{s0}$ is the shear damage initiation displacement; $\delta_{sf}$ is the shear failure displacement; and D is the damage factor, which can be calculated through $D=\frac{(\left|\delta_{s}\right|-\delta_{s0})\delta_{sf}}{(\delta_{sf}-\delta_{s0})\left|\delta_{s}\right|}$.

(2) Mixed-mode damage relation

The quadric criterion [31,32,33] was adopted to define the initiation of the damage process in the mixed-mode condition, and its expression can be expressed as follows:

(3) $${\left(\frac{t_{n}}{t_{n0}}\right)}^{2}+{\left(\frac{t_{s}}{t_{s0}}\right)}^{2}=1$$

where $t_{n0}$ is the normal strength and $t_{s0}$ is the shear strength.

The PL criterion defined the evolution of the damage, and it can be given as follows:

(4) $$\frac{G_{n}^{r}}{G_{n}}+\frac{G_{s}^{r}}{G_{s}}=1$$

where $G_{n}$ is the normal fracture energy and $G_{s}$ is the shear fracture energy; these two fracture energies can be calculated through the geometry relation in Figure 8. $G_{n}^{r}$ is the normal energy release rate in the mixed-mode condition and $G_{s}^{r}$ is the shear energy release rate in the mixed-mode condition. These four parameters can be expressed as follows:

(5) $$\left\{\begin{array}{l} G_{n}=\frac{k_{n}\delta_{n0}\delta_{nf}}{2}\\ G_{s}=\frac{k_{s}\delta_{s0}\delta_{sf}}{2} \end{array}\right.\;,\;\left\{\begin{array}{l} G_{n}^{r}=\frac{k_{n}\delta_{n0}^{r}\delta_{nf}^{r}}{2}\\ G_{s}^{r}=\frac{k_{s}\delta_{s0}^{r}\delta_{sf}^{r}}{2} \end{array}\right.$$

where $\delta_{n0}^{r}$ is the normal relative damage initial displacement in the mixed-mode condition; $\delta_{nf}^{r}$ is the normal relative failure displacement in the mixed-mode state; $\delta_{s0}^{r}$ is the shear relative damage initial displacement in the mixed-mode condition; and $\delta_{sf}^{r}$ is the shear relative failure displacement in the mixed-mode condition.

Assuming that the loading path is monotonous, by substituting $\frac{\langle \delta_{n}\rangle }{\delta_{n0}^{r}}=\frac{\left|\delta_{s}\right|}{\delta_{s0}^{r}}$ (<> are the Macaulay brackets, and this equation is suitable for the condition when $\delta_{n}>0$), the geometry relation in Figure 8, $t_{n}=k_{n}\delta_{n0}^{r}$, and $t_{s}=k_{s}\delta_{s0}^{r}$ into Equation (3), the relative damage initial displacements can be given as follows:

(6) $$\left\{\begin{array}{l} \delta_{n0}^{r}=\frac{\langle \delta_{n}\rangle \delta_{n0}\delta_{s0}}{\sqrt{\delta_{n0}{}^{2}\delta_{s}{}^{2}+\delta_{n}{}^{2}\delta_{s0}{}^{2}}}\\ \delta_{s0}^{r}=\frac{\left|\delta_{s}\right|\delta_{n0}\delta_{s0}}{\sqrt{\delta_{n0}{}^{2}\delta_{s}{}^{2}+\delta_{n}{}^{2}\delta_{s0}{}^{2}}} \end{array}\right.$$

Additionally, by substituting $\frac{\delta_{n0}^{r}}{\delta_{nf}^{r}}=\frac{\delta_{s0}^{r}}{\delta_{sf}^{r}}$ (monotonous loading path) and Equation (5) into Equation (4), the failure displacements can also be given as follows:

(7) $$\left\{\begin{array}{l} \delta_{nf}^{r}=\frac{2\delta_{n0}^{r}G_{n}G_{s}}{k_{n}\delta_{n0}^{r}{}^{2}G_{s}+k_{s}\delta_{s0}^{r}{}^{2}G_{n}}\\ \delta_{sf}^{r}=\frac{2\delta_{s0}^{r}G_{n}G_{s}}{k_{n}\delta_{n0}^{r}{}^{2}G_{s}+k_{s}\delta_{s0}^{r}{}^{2}G_{n}} \end{array}\right.$$

Based on these calculated parameters, the total displacement δ, total initial damage displacement $\delta_{0}$, and total failure displacement $\delta_{f}$ can be calculated as follows:

(8) $$\left\{\begin{array}{l} \delta =\sqrt{{\langle \delta_{n}\rangle }^{2}+\delta_{s}^{2}}\\ \delta_{0}=\sqrt{\delta_{n0}^{r}{}^{2}+\delta_{s0}^{r}{}^{2}}\\ \delta_{f}=\sqrt{\delta_{nf}^{r}{}^{2}+\delta_{sf}^{r}{}^{2}} \end{array}\right.$$

Hence, the damage factor in the mixed-mode condition can be finally given as follows:

(9) $$D=\frac{(\delta -\delta_{0})\delta_{f}}{(\delta_{f}-\delta_{0})\delta }$$

(3) Friction effect

When the interface is damaged, friction occurs at the interface when the interface is closed. For this reason, the friction effect should be considered precisely. In this constitutive model, the fiction stress is calculated according to the interfacial sliding condition.

(a) Interfacial not sliding

In this condition, the friction stress $T_{f}$ can be calculated as follows:

(10) $$T_{f}=k_{s}(\delta_{s}-\delta_{s}^{slide})\;(k_{s}(\delta_{s}-\delta_{s}^{slide})\leq T_{f}{}_{\max})$$

where $\delta_{s}^{slide}$ is the sliding displacement which has been generated and $T_{f}{}_{\max}$ is the maximum static friction stress, which can be given according to the friction law:

(11) $$T_{f}{}_{\max}=f\langle -k_{n}\delta_{n}\rangle$$

where f is the friction coefficient.

(b) Interfacial sliding

In this condition, the friction stress equals the maximum static friction stress. In addition, the corresponding interfacial sliding displacement should be updated:

(12) $$\left\{\begin{array}{l} T_{f}=f\langle -k_{n}\delta_{n}\rangle \frac{\delta_{s}-\delta_{s}^{slide}}{\left|\delta_{s}-\delta_{s}^{slide}\right|}\\ \delta_{s}^{slide*}=\delta_{s}-\frac{T_{f}}{k_{s}} \end{array}\right.(k_{s}\left|\delta_{s}-\delta_{s}^{slide}\right|>T_{f}{}_{\max})$$

where $\delta_{s}^{slide*}$ is the updated interfacial sliding displacement.

(4) Stresses in the mixed model

Finally, combining the damage relation and friction effect, the stresses can be given as follows:

(13) $$t_{n}=\left\{\begin{array}{ll} k_{n}\delta_{n} & \delta \leq \delta_{0}\;\mathrm{or}\;\delta_{n}\leq 0\;\\ (1-D)k_{n}\delta_{n} & \delta_{0}<\delta <\delta_{f}\\ 0 & \delta >\delta_{f} \end{array}\right.$$

(14) $$t_{s}=\left\{\begin{array}{ll} k_{s}\delta_{s} & \left|\delta_{s}\right|\leq \delta_{s0}\\ (1-D)k_{s}\delta_{s}+D\cdot T_{f}\; & \delta_{s0}<\left|\delta_{s}\right|<\delta_{sf}\\ D\cdot T_{f} & \left|\delta_{s}\right|\geq \delta_{sf} \end{array}\right.$$

(5) Internal energy calculation

According to the stresses defined above, three corresponding kinds of energy can be extracted to analyze the internal fracture behavior, which are: (1) the normal stress work $E_{n}$; (2) the shear stress work $E_{s}$; and (3) the friction stress work $E_{f}$. These energies can be calculated by:

(15) $$\begin{array}{l} E_{n}=\sum_{\mathrm{Cohesive}\;\mathrm{elements}}t\int_{l}(\int_{0}^{\delta_{n}}t_{n}d\delta )dl\;\;,\\ E_{s}=\sum_{\mathrm{Cohesive}\;\mathrm{elements}}t\int_{l}(\int_{0}^{\delta_{s}}(t_{s}-D\cdot T_{f})d\delta )dl\;,\\ E_{f}=\sum_{\mathrm{Cohesive}\;\mathrm{elements}}t\int_{l}(\int_{0}^{\delta_{s}}D\cdot T_{f}\cdot d\delta )dl \end{array}$$

where l is the length of the zero-thickness cohesive element and t is the calculation thickness.

## 4. Numerical Analysis and Discussion of the TPB Experiments

### 4.1. Input Data of the Finite Element Model

**Figure 9:**
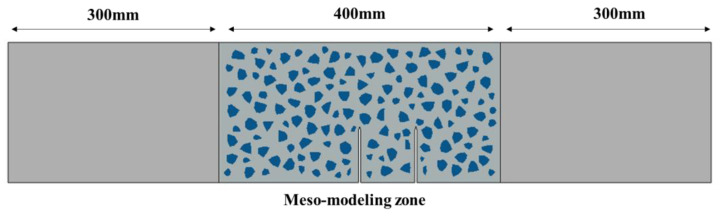
Numerical model of the TPB specimens.

The meso-model was used to analyze the concrete double-crack problem, as shown in Figure 9. In the fracture process zone, the meso-model was used to characterize the fracture behavior of concrete, while in the non-fracture zone, the macro-model was used to reflect the elastic behavior of concrete. The meso-modeling zone and the macro-modeling zone were tied together on their boundary. Through repeated trials, the length of the meso-modeling zone was set to 400 mm, and this area can completely characterize the fracture behavior of the specimen.

**Figure 10:**
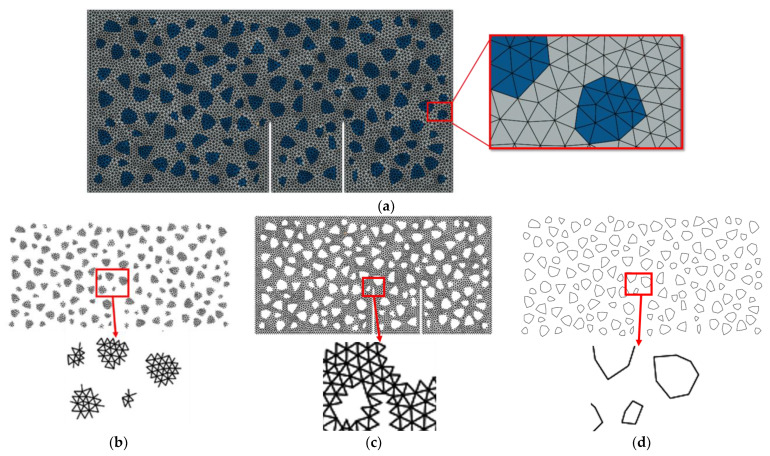
Mesh of the meso-modeling zone: (**a**) general view; (**b**) the zero-thickness elements of aggregates CE_AGG; (**c**) the zero-thickness elements of mortar CE_MOR; (**d**) the zero-thickness elements of ITZ CE_ITZ.

The mesh of the numerical model is shown in Figure 10. Through the mesh independence test, the element size was set to 3 mm. One typical model (meso-modeling zone) contains about 50,000 nodes, 17,000 solid elements, and 25,000 zero-thickness cohesive elements.

According to the experimental results, repeat trial computations, and the previous works on cohesive elements [29,30,31,32,33,34], the material parameters (meso-modeling zone) of cohesive elements were determined and are listed in Table 3, assuming the aggregate will not crack. In addition, for the meso-modeling zone, the elastic moduli of the mortar and aggregate were 40 GPa and 60 GPa, respectively, and the corresponding Poisson ratios were set to 0.22. For the macro-modeling zone, the elastic modulus and Poisson’s ratio were 45 GPa and 0.22, respectively. All specimens are subjected to a concentrated displacement load at the top of the midspan, and the final displacement value was 0.5 mm.

The numerical models were solved in the ABAQUS/EXPLICIT solver [36], with the user subroutine VUMAT in which the proposed constitutive model was implemented. The loading time was set to 1 s to ensure a quasi-static loading condition. Three random numerical specimens were calculated in each experiment group to eliminate accidental error. Through computation, all numerical models in the same group showed a very similar result. For this reason, in each group, only one typical numerical specimen was chosen to discuss in this study.

### 4.2. Fracture Behavior of the Standard and Double-Crack Beams

**Figure 11:**
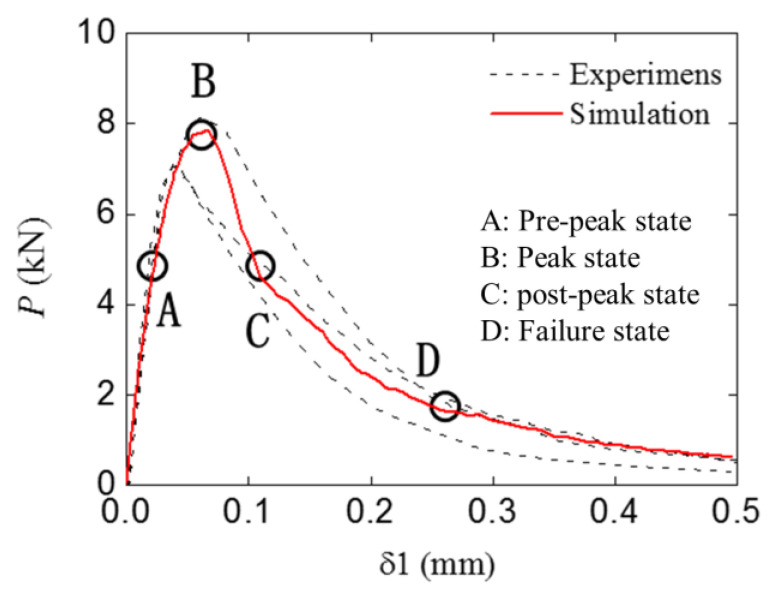
The $P-\delta_{1}$ curve of the standard group with the simulation result.

The standard group’s numerical and experimental P-δ1 curves are shown in Figure 11. The numerical results fit well with the experimental ones in terms of the curve shape and amplitude. To further investigate the fracture process and corresponding stress distribution, four typical states (pre-peak state, peak state, post-peak state, and failure state) were chosen in Figure 9, and the four states were marked by A to D.

**Figure 12:**
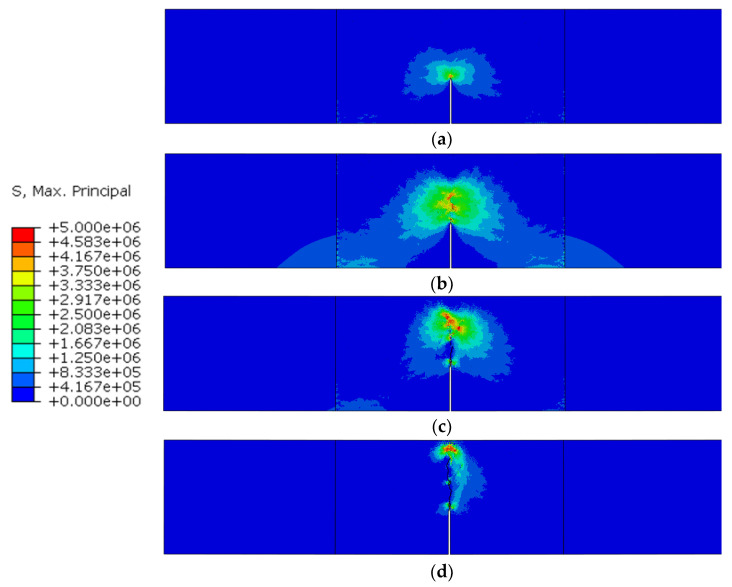
Maximum principal stress distribution in different states marked in Figure 11: (**a**) state A; (**b**) state B; (**c**) state C; (**d**) state D.

The distribution of maximum stress during the fracture process is shown in Figure 12, and the cracks are represented by deleting the cohesive elements whose damage factor is equal to 1. The stress distribution regular pattern can be summarized as follows: (1) In the early stage of the fracture process, a stress concentration zone exists in the crack tip. (2) In the crack-propagation stage, the damaged area extends upward. Although the damaged area does not turn into a macro-crack, the stress concentration zone moves to the tip of the damaged area, which means the stress concentration zone moves further than the crack. (3) In the final stage, the damaged area extends to the top of the specimen, and the bearing capacity of the specimen is almost lost.

It should be noted that the crack does not extend along an ideal straight line due to the random distribution of aggregate. As a result, some fracture surfaces may not separate completely. Thus, a large stress still exists in some areas without complete damage, as shown in Figure 12c,d.

**Figure 13:**
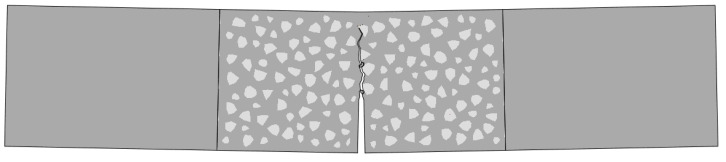
Fracture pattern of the standard specimen.

The final fracture pattern of the standard beam is shown in Figure 13, and the deformation shape has been magnified 15 times. Due to the low strength of the ITZ, the crack will preferentially pass through a nearby ITZ in the fracture process. This regular pattern leads to the randomness of the fracture propagation path, which makes the simulation results closer to the experimental ones compared to the idealized numerical macro-model.

**Figure 14:**
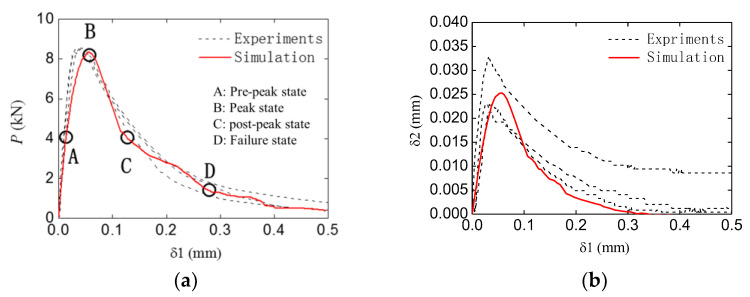
Typical simulation $P-\delta_{1}$ and $\delta_{2}-\delta_{1}$ curves of the double-crack beam (TPBSC80 or TPBCD80): (**a**) $P-\delta_{1}$ curve; (**b**) $\delta_{2}-\delta_{1}$ curve.

The typical (the group TPBSC80 or TPBCD80) numerical and experimental $P-\delta_{1}$ and $\delta_{2}-\delta_{1}$ curves of a double-crack beam are shown in Figure 14. It can be seen that the numerical results still show an agreement with the experimental ones. As was the case with the standard group, four typical states were chosen to investigate the fracture process of the specimen. The four states are marked by A to D, as shown in Figure 14a.

**Figure 15:**
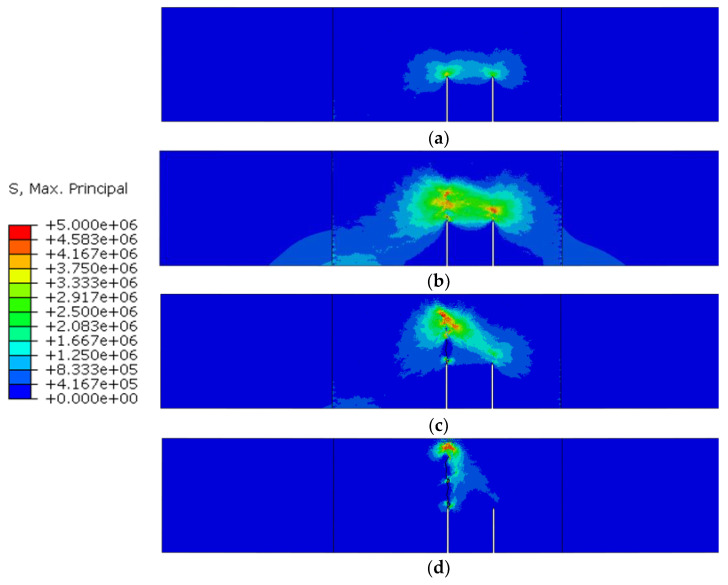
Maximum principal stress nephogram of the different stages in Figure 14: (**a**) stage A; (**b**) stage B; (**c**) stage C; (**d**) stage D.

The maximum stress distribution of the double-crack beam (the group TPBSC80 or TPBCD80) during the fracture process is shown in Figure 15. The corresponding regular evolution of the stress distribution can be summarized as follows: (1) In the early stage of the fracture process, there is a two-stress concentration zone at both crack tips, and the stress value of the main crack is larger than the other one. (2) In the stable crack propagation stage, both stress concentration zones extend upward, and the stress concentration zone extending from the main crack moves further than one extending from the second crack. (3) In the unstable crack propagation stage (post-peak stage), the stress concentration zone of the second crack gradually vanishes, which means the second crack stops propagating and gradually closes. Meanwhile, the main crack keeps propagating. (4) In the final stage, the damaged area originating from the main crack extends to the top of the specimen, and the bearing capacity of the specimen is almost lost.

**Figure 16:**
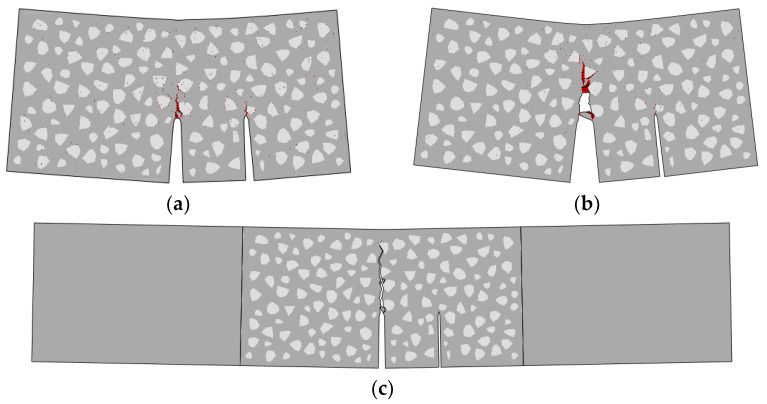
Fracture pattern of a double-crack specimen (the group TPBSC80 or TPBCD80): (**a**) pre-peak stage; (**b**) post-peak stage; (**c**) the final fracture pattern.

To obtain more details of the fracture process of the double-crack specimen (the group TPBSC80 or TPBCD80), the deformation of the beam at the pre-peak stage (Figure 16a) and the post-peak stage (Figure 16b) was extracted, and the damaged areas were marked in red. It can be seen that at the pre-peak stage, both CMODs open and both the initial cracks extend. In the post-peak stage, the second crack closes and the main crack continues to open.

**Figure 17:**
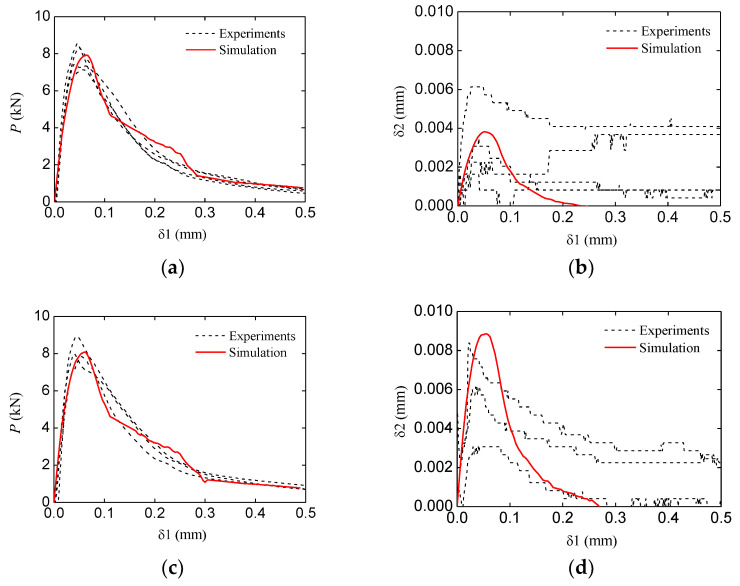

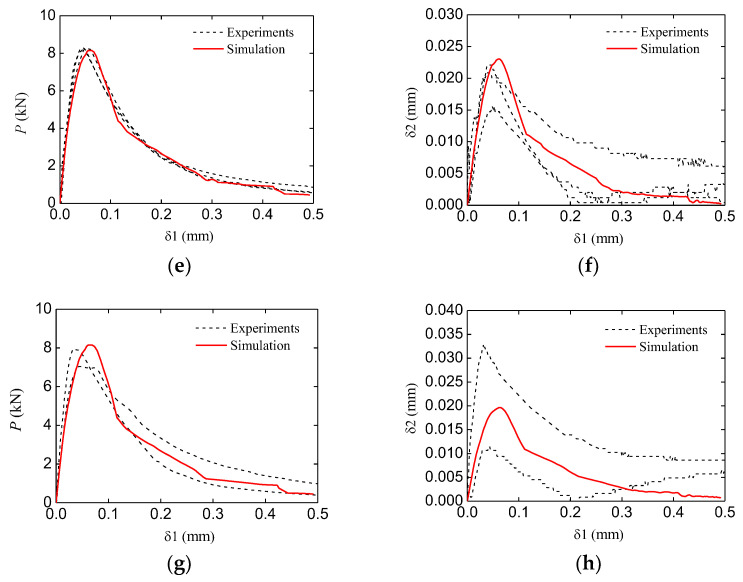
Comparison of experiments and simulation results: (**a**,**b**) $P-\delta_{1}$ and $\delta_{2}-\delta_{1}$ curves of the TPBSC40 group; (**c**,**d**) $P-\delta_{1}$ and $\delta_{2}-\delta_{1}$ curves of the TPBSC6 group 0; (**e**,**f**) $P-\delta_{1}$ and $\delta_{2}-\delta_{1}$ curves of the TPBCD120 group; (**g**,**h**) $P-\delta_{1}$ and $\delta_{2}-\delta_{1}$ curves of the TPBCD160 group.

The simulation and experiment comparisons of the other groups (the $P-\delta_{1}$ curves and the $\delta_{2}-\delta_{1}$ curves) are shown in Figure 17. The comparison results indicate that the numerical meso-model adopted in this study can appropriately characterize the fracture behavior of concrete with multiple cracks.

### 4.3. Bearing Capacity Analysis

**Figure 18:**
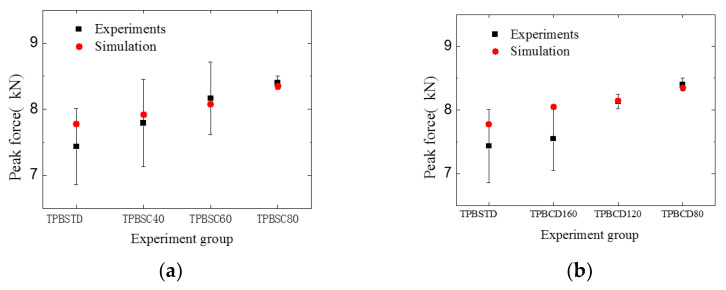
Comparison of the peak force between different experiment groups: (**a**) different second crack lengths; (**b**) different crack distances.

To investigate the impact of the two cracks on the specimen’s bearing capacity, first, the relation between peak force and different experiment groups was analyzed, as shown in Figure 18. The figure indicates that the peak force of the double-crack specimens is a bit larger than that of the standard specimens. Similar to the experimental results, with the increase in the second crack’s length or with the decrease in the crack distance, the peak force of the beam increases correspondingly, although the increase is minimal. To reveal why multiple cracks can slightly enhance the bearing capacity of the concrete specimen, an energy analysis was carried out based on the meso-model.

### 4.4. Energy Analysis

**Figure 19:**
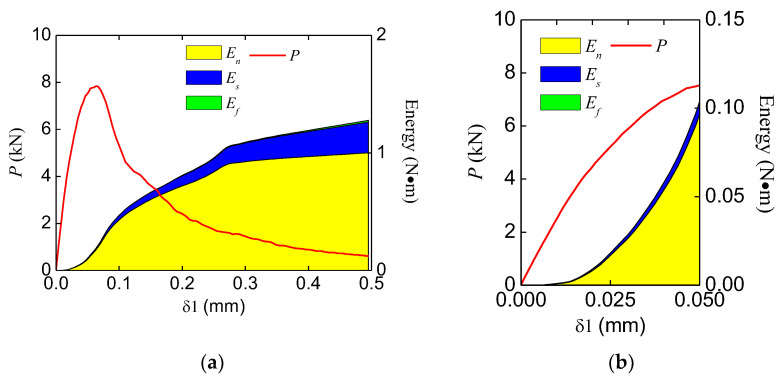
Typical evolution of the internal energy (TPBSTD) in (**a**) the whole fracture process; (**b**) the pre-peak stage.

Through the analysis, the energy consumption in the double-crack beams was very similar to the standard beam. The typical energy evolution of a standard beam is shown in Figure 19. During the fracture process, the normal stress work (mode I fracture) dominates the energy consumption, and a small amount of shear stress work (mode II fracture) also exists. In addition, friction has little effect in this study. Thus, the whole fracture process is a composite fracture process dominated by mode I fractures. To investigate the relation between the peak force and the proportion of the energy increase at the pre-peak stage, the energy increases of different groups were extracted and are listed in Table 4.

**Figure 20:**
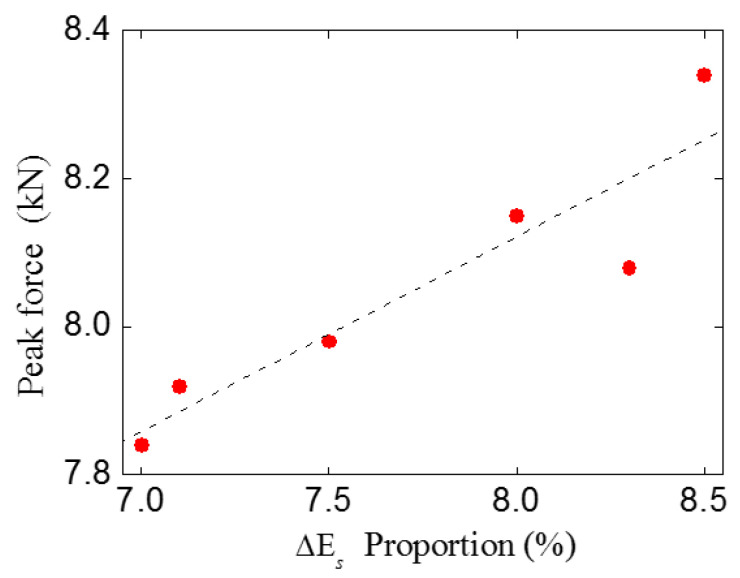
Relation between peak force and $\Delta E_{s}$ proportion.

Through a comparison, it can be found that as the proportion of shear stress work (mode II fractures) increases, the peak force increases correspondingly. The peak force is highly relevant to the shear stress work proportion, and a linear relationship exists between these two parameters, as shown in Figure 20. Thus, it can be inferred that due to the existence of multiple cracks, more shear stress work will be consumed during the fracture process. For concrete material, the shear strength and corresponding fracture energy are much larger than the tensile ones. For this reason, in this study, the bearing capacity of the double-crack beams was slightly higher than the standard beams.

**Figure 21:**
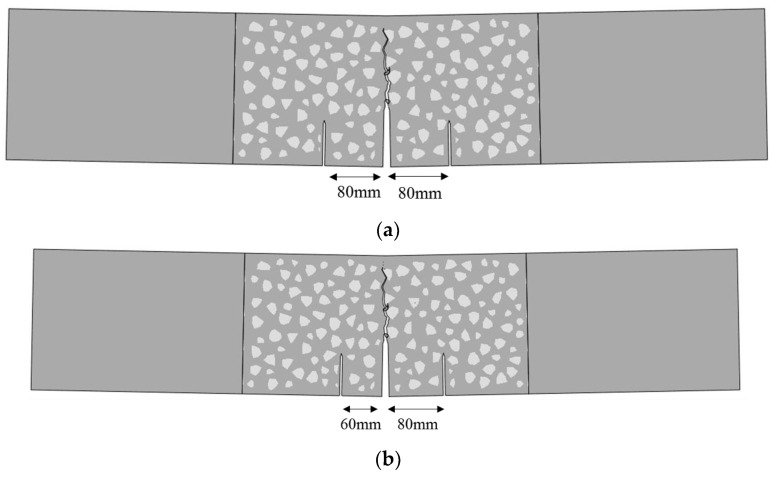
The fracture pattern of three-crack specimens with (**a**) a symmetrical crack distribution; (**b**) an asymmetrical crack distribution.

To validate the statement above, a group of numerical tests with three parallel cracks (symmetrical and asymmetrical distributions of cracks) were also carried out, and the fracture paths are shown in Figure 21. The bearing capacity of three-crack specimens (8.03 kN, 8.04 kN, respectively) is still higher than the standard one (7.84 kN), regardless of whether the distribution of the crack is symmetrical or asymmetrical.

## 5. Conclusions

In this paper, a series of TPB experiments were carried out to investigate the influence of multiple cracks on concrete fracture behavior. Six groups of double-crack TPB beams were designed and tested. A numerical meso-model was developed to analyze the fracture behavior of double-crack concrete specimens. In this meso-model, cohesive elements were adopted to characterize the potential fracture surfaces, and the corresponding constitutive model combining the damage relation and friction effect was developed. Through comparison, all the numerical results agreed with the experimental ones. Based on this model, the influence of multiple parallel cracks on the bearing capacity of concrete was studied by analyzing the energy consumption. The conclusions can be summarized as follows:

1. In the mode Ⅰ fracture (or composite fracture dominated by mode Ⅰ fracture) condition, multiple cracks in a small zone will slightly increase the bearing capacity of the concrete. With an increase in the other crack’s lengths or with a decrease in the distance between cracks, the bearing capacity increases.
2. In terms of energy consumption, the proportion of shear stress work (mode II) is highly relevant to the bearing capacity of multiple-parallel-crack concrete. Multiple parallel cracks change the proportion of mode Ⅱ fractures and finally cause an increase in the concrete bearing capacity.

Based on the conclusions above, in the mode Ⅰ fracture (or composite fracture dominated by mode Ⅰ fracture) condition, multiple cracks in a small zone can be equally considered as one crack from the perspective of safety design.

It should be noted that in this study, only the concrete fracture behavior with multiple parallel cracks was investigated. However, in practical engineering applications, the distribution of cracks is more random and the fracture mode is more complex. Therefore, in subsequent studies, it is necessary to further analyze the mechanical behavior of multiple cracks under a mixed-mode (mode I and mode II) fracture process.

## Figures and Tables

**Table 1:** The geometry information of double-crack concrete TPB beams.

Specimens	Main Crack Length a0/mm	Second Crack Length a1/mm	Cracks Distance d/mm
TPBSTD-1~4	80	—	
TPBSC40-1~4	80	40	80
TPBSC60-1~4	80	60	80
TPBSC80-1~4 (TPBCD80-1~4)	80	80	80
TPBCD120-1~4	80	80	120
TPBCD160-1~4	80	80	160

**Table 2:** Summary of experimental results.

Specimens	Peak Load (kN)	Corresponding CMOD $\delta_{1}$ (mm)	Corresponding CMOD $\delta_{2}$ (mm)
TPBSTD-1	7.02	0.0388	—
TPBSTD-2	7.04	0.0387	—
TPBSTD-4	8.07	0.0613	—
Average	7.38	0.0462	
TPBSC40-1	7.37	0.0387	0.0016
TPBSC40-2	8.48	0.0463	0.0061
TPBSC40-3	8.26	0.0500	0.0031
TPBSC40-4	7.07	0.0375	0.0017
Average	7.80	0.0431	0.0031
TPBSC60-1	8.82	0.0463	0.0057
TPBSC60-2	7.78	0.0525	0.0031
TPBSC60-3	7.91	0.0400	0.0076
Average	8.17	0.0463	0.0055
TPBSC80-1	8.39	0.0363	0.0213
TPBSC80-2	8.48	0.0450	0.0221
TPBSC80-3	8.49	0.0337	0.0324
Average	8.45	0.0383	0.0253
TPBCD120-1	8.21	0.0413	0.0208
TPBCD120-2	8.03	0.0450	0.0221
TPBCD120-3	8.20	0.0537	0.0147
Average	8.15	0.0467	0.0192
TPBCD160-2	7.09	0.0450	0.0106
TPBCD160-4	7.83	0.0350	0.0319
Average	7.46	0.0400	0.0213

**Table 3:** Material parameters applied to the concrete.

Element Type	$k_{n}$$,k_{s}$ (GPa/m)	$t_{n0}$ (MPa)	$t_{s0}$ (MPa)	$G_{n0}$ (N/m)	$G_{s0}$ (N/m)	f
CE_MOR	106	4.2	14.7	70	700	0.35
CE_ITZ	106	2.1	7.35	35	350	0.35
CE_AGG	106	-	-	-	-	-

**Table 4:** Energy increments of different double cracks specimens.

Experiment Group	Peak Force (kN)	$\Delta E_{n}$ (N∙m)	$\Delta E_{s}$ (N∙m)	Total Energy Increment (N∙m)	$\Delta E_{n}$ Proportion (%)	$\Delta E_{s}$ Proportion (%)
Standard TPBSTD	7.84	0.156	0.012	0.167	93.0	7.0
TPBSC40	7.92	0.163	0.013	0.176	92.9	7.1
TPBSC60	8.08	0.144	0.013	0.157	91.7	8.3
TPBSC80 (TPBCD80)	8.34	0.151	0.014	0.165	91.5	8.5
TPBSC120	8.15	0.145	0.013	0.158	92.0	8.0
TPBSC160	7.98	0.141	0.012	0.153	92.5	7.5

## Data Availability

Not applicable.

## Abbreviations

D	Damage factor in the mode-Ⅰ, mode-Ⅱ, and mixed-mode conditions
$F_{t}$	Tensile strength of the interface
$F_{t}^{r}$	Relative tensile strength of the interface
*f*	Friction coefficient
$G_{Ⅰ}$	Fracture energy in the mode-Ⅰ condition
$G_{Ⅱ}$	Fracture energy in the mode-Ⅱ condition
$G_{Ⅰ}^{r}$	Energy release rate in the normal direction
$G_{Ⅱ}^{r}$	Energy release rate in the tangential direction
$k_{n}$	Interface stiffness in the normal direction
$T_{f}$	Friction stresses in the tangential direction
$T_{f}{}_{\max}$	Maximum friction stress
σ	Normal stress
τ	Shear stress
$\tau_{0}$	Shear strength
$\tau_{0}^{r}$	Relative shear strength
$\delta_{n}$	Displacement in the normal direction
$\delta_{n0}$	Normal displacement at the onset of interfacial softening in the mode-Ⅰ condition
$\delta_{n0}^{r}$	Relative normal displacement at the onset of interfacial softening in the mixed-mode condition
$\delta_{nf}$	Normal displacement at the onset of interfacial failure in the mode-Ⅰ condition
$\delta_{nf}^{r}$	Relative normal displacement at the onset of interfacial failure in the mixed-mode condition
$\delta_{s}$	Total displacement in the tangential direction
$\delta_{s0}$	Tangential displacement at the onset of interfacial softening in the mode-Ⅱ/Ⅲ condition
$\delta_{s0}^{r}$	Relative tangential displacement at the onset of interfacial softening in the mixed-mode condition
$\delta_{sf}$	Tangential displacement at the onset of interfacial failure in the mode-Ⅱ/Ⅲ condition
$\delta_{sf}^{r}$	Relative tangential displacement at the onset of interfacial failure in the mixed-mode condition
$\delta_{s}^{slide}$	Tangential sliding displacement that has been generated during the loading process
δ	Total relative displacement
$\delta_{0}$	Total relative displacement at the onset of interfacial softening in the mixed-mode condition
$\delta_{f}$	Total relative displacement at the onset of interfacial failure in the mixed-mode condition
TPB	Three-point bending
CMOD	Crack mouth opening displacement
CZM	Cohesive zone model
